# Clinical features and treatment of pediatric patients with drug-induced anaphylaxis: a study based on pharmacovigilance data

**DOI:** 10.1007/s00431-017-3048-z

**Published:** 2017-11-22

**Authors:** Yan Xing, Hua Zhang, Shusen Sun, Xiang Ma, Roy A. Pleasants, Huilin Tang, Hangci Zheng, Suodi Zhai, Tiansheng Wang

**Affiliations:** 1 Department of Pediatrics, Division of Pulmonology & Allergy, Peking University Third Hospital, Beijing, China; 2 Research Center of Clinical Epidemiology, Peking University Third Hospital, Beijing, China; 3 College of Pharmacy and Health Sciences, Western New England University, Springfield, MA USA; 4 Department of Pharmacy, Peking University Third Hospital, Beijing, China; 5 Division of Pulmonary, Allergy, and Critical Care Medicine, Duke University Asthma, Allergy, and Airways Center, Durham, NC USA; 6 Richard M. Fairbanks School of Public Health, Department of Epidemiology, Indiana University–Purdue University Indianapolis, Indianapolis, IN USA; 7 Institute for Drug Evaluation, Peking University Health Science Center, Beijing, China; 8 Department of Epidemiology, Gillings School of Global Public Health, University of North Carolina at Chapel Hill, Chapel Hill, NC USA

**Keywords:** Children, Epinephrine, Signs and symptoms, Drug-induced anaphylaxis

## Abstract

We assessed the clinical features and treatment of pediatric patients with drug-induced anaphylaxis in clinical settings. Pediatric drug-induced anaphylaxis cases collected by the Beijing Pharmacovigilance Database from 2004 to 2014 were analyzed. A total of 91 cases were identified. Drug-induced anaphylaxis was primarily caused by antibiotics (53%). Children of 0–5 years were more likely to develop cyanosis symptoms than children of 13–17 years (OR = 5.14, 95%CI [1.74, 15.20], *P* = 0.002). Children of 13–17 years were more likely to develop hypotension than children of 6–12 years (OR = 11.79, 95%CI [2.28, 60.87], *P* = 0.002), and to manifest both neurological symptoms (OR = 3.56, 95%CI [1.26, 10.08], *P* = 0.015) and severe anaphylaxis than children of 0–5 years (OR = 15.46, 95%CI [1.85, 129.33], P = 0.002). Supratherapeutic doses of epinephrine were more likely with intravenous (IV) bolus (92%) in contrast to either intramuscular (IM) (36%, OR = 19.25, 95%CI [1.77, 209.55], *P* = 0.009) or subcutaneous (SC) injections (36%, OR = 19.80, 95% CI [1.94, 201.63], *P* = 0.005). Only 62 (68%) patients received epinephrine treatment as the first-line therapy.

*Conclusion*: This study demonstrates that antibiotics were the most common cause of pediatric drug-induced anaphylaxis. Children may present with different anaphylactic signs/symptoms based on age groups. Epinephrine is under-utilized and provider education on the proper management of drug-induced anaphylaxis is warranted.

**Table:** 

**What is Known:** • *The most common causes of anaphylaxis in children are allergies to foods. Drugs are the second most common cause of pediatric anaphylaxis.* *• IM epinephrine is the recommended initial treatment of anaphylaxis.*
**What is New:** • *Drug-induced anaphylaxis in pediatric patients has age-related clinical features.* *• IV bolus epinephrine was overused and associated with supratherapeutic dosing.*

**Electronic supplementary material:**

The online version of this article (10.1007/s00431-017-3048-z) contains supplementary material, which is available to authorized users.

## Introduction

Anaphylaxis, the most severe manifestation of an acute allergic reaction, is a medical emergency and in rare cases may cause death [25]. It typically involves two or more body systems including skin and/or mucosa, upper and lower respiratory tract, gastrointestinal tract, cardiovascular system, and central nervous system [25]. First-line treatment for anaphylaxis is intramuscular (IM) epinephrine injection in the anterolateral thigh [25]. Pediatric emergency department (ED) visits for children with anaphylaxis increased from 5.7 to 11.7 per 10,000 visits from 2009 to 2013 in the USA [14, 20], and this has become an important health issue. The most common causes of anaphylaxis in children are allergies to foods, followed by medications, stinging insect venom, blood products, immunotherapy, latex, vaccines, and contrast media [13, 33].

Despite drug-induced anaphylaxis (DIA) being the second most common cause of pediatric anaphylaxis [12], information on its characteristics and management is limited, and data on DIA in children outside the USA and Europe is sparse. Here, we present an analysis of 91 pediatric DIA cases based on data extracted from the Beijing Pharmacovigilance Database (BPD) from 2004 to 2014. The purpose of the study was to evaluate the elicitors, describe the main clinical manifestations of anaphylaxis and treatment of DIA in pediatric patients.

## Materials and methods

### Study participants

This study was considered to be exempt from further review by the Institutional Review Board, Peking University Third Hospital. We utilized the BPD provided by the Beijing Center for Adverse Drug Reaction Monitoring (BCADR) for this study. A total of 94 participating hospitals in the Beijing region are required to report severe adverse drug event (ADE) cases to this database. Cases are self-reported to the respective pharmacy department by physicians, nurses, and pharmacists. The BPD is created to collect well-defined and standardized data of affected patients experiencing ADEs in the Beijing region of China. We identified all patients with a reported drug-induced acute allergic reaction or anaphylaxis using the following search terms: “anaphylaxis,” “anaphylactic shock,” “allergy,” “allergic reaction,” and “hypersensitivity” from the BPD database for the period covering January 1, 2004 to December 31, 2014. A data extraction form was developed to validate the diagnosis and severity of anaphylaxis and the use of epinephrine. The form was tested and revised based on a pilot analysis of database records from 250 patients. Two trained extractors performed the extraction independently to ensure consistency, and discrepancies were resolved by an experienced investigator with expertise in standardized medical record extraction. Two physician adjudicators independently determined the diagnosis of anaphylaxis and assessed the case severity based on the information extracted from each case. All disagreements were resolved through discussions. We divided the severity of each anaphylaxis case into either mild to moderate or severe categories (Appendix 1) [3]. The information on BCADR, detailed case query strategy and data extraction from our research team has been previously published [32, 34], in which a cohort of 1189 DIA patients were identified. A subgroup of pediatric patients (less than 18 years of age) from the base cohort was included in this analysis.

### Study design

Patient’s age was categorized into three strata: 0–5, 6–12, and 13–17 years. We investigated differences between groups with regards to admission diagnosis, suspected elicitors, organ systems’ involvement, and therapies to treat anaphylaxis. We analyzed the dose and the administration route (intravenous IV, IM, and subcutaneous SC) of epinephrine. To analyze the supratherapeutic dosing of epinephrine, initial pediatric epinephrine dosing was categorized into non-supratherapeutic or supratherapeutic [11, 17] as determined by weight-based dosing (≤ 0.01 mg/kg vs > 0.01 mg/kg of 1:1000 solution with a maximum dose of 0.5 mg for IM and SC routes, and or 0.001 mg/kg for IV bolus with a maximum dose of 0.1 mg of 1:10,000 solution). When weights were not available, we assessed the pediatric IM/SC epinephrine dosing based on age groups (<6, 6–12, and >12 years) [28], and for IV bolus we used age to estimate body weight [31] to calculate the dose. We did not analyze supratherapeutic dosing with epinephrine IV continuous infusion as it is usually titrated to clinical effects, and not all the necessary information for calculating the total dose was available, such as the exact time for dose modifications.

We compared the baseline information, anaphylactic symptoms, outcome for patients receiving epinephrine monotherapy, corticosteroid monotherapy, as well as epinephrine and corticosteroid combination therapy. We also compared outcomes between patients who received epinephrine as an initial treatment and patients who did not receive it as an initial treatment.

### Statistical analyses

The statistical analysis was performed using the SPSS version 18 (SPSS Inc., IL, USA). Dosing data across age groups were expressed as median (min, max) and compared using the Kruskal-Wallis test, depending on non-normality. If the *P* value of the Kruskal-Wallis test is less than 0.05, the Wilcoxon rank sum test was conducted for pairwise comparisons between groups. The dichotomous variables were described as frequency (percentage) and differences from age groups were compared using the Pearson’s chi-square test (when no cell in the table have an expected count less than 1, and no more than 20% of the cells should have an expected count less than 5) or the Fisher’s exact test. The *P* values of pairwise comparisons were adjusted by the Bonferroni correction for multiple comparisons. All tests were two-tailed tests, where a difference with *P* < 0.05 was considered to be statistically significant.

#### Data availability

The data that support the findings of this study are available from the BCADR, but restrictions apply to the availability of these data, which were used under license for the current study, and so are not publicly available. Data are, however, available from the authors upon reasonable request and with permission from the BCADR.

## Results

### Patients and anaphylaxis elicitors

A total of 9425 patients with drug-induced hypersensitivity reactions were identified from the BPD using our search terms, and 91 pediatric patients were ultimately included in our analysis (flowchart is shown in Appendix 2). The majority of patients (88, 97%) developed anaphylaxis during their hospitalizations, and 3 patients presented to an ED. Of the 88 inpatients, the number of patients who received anaphylaxis treatment in EDs and non-ED settings were 11(13%) and 77(88%), respectively. Of the 91 patients, 35% were 0–5 year old, 26% were 6–12 year old, and 39% were 13–17 year old; and 62% of the patients were males (Table 1). There were no significant differences for admission diagnoses and elicitors among the three age groups, except for a significant difference between the 6–12 years group and 13–17 years group for biologics use (*P* = 0.032).

**Table 1:** Demographics, original diseases, and elicitors of DIA in children by age group

	All no. (%) (*n* = 91)	0–5 years no. (%) (*n* = 32)	6–12 years no. (%) (*n* = 24)	13–17 years no. (%) (*n* = 35)	*χ* ^2^	*P* *
Demographics						
Male	56(62)	20(63)	11(46)	25(71)	3.960	0.138
Female	35(39)	12(38)	13(54)	10(29)		
Admission diagnoses						
Infectious	49(54)	20(63)	12(50)	17(49)	1.499	0.473
RTI	38(42)	18(56)	8(33)	12(34)	4.267	0.118
Stress/operation/trauma	15(17)	3(9)	2(8)	10(29)		0.061
Tumor	10(11)	4(13)	3(13)	3(9)		0.837
Gastrointestinal disease	8(9)	3(9)	2(8)	3(9)		1.000
Rheumatological disease	4(4)	2(6)	2(8)	0(0)		0.184
Asthma	1(1)	0(0)	1(4)	0(0)		0.264
Cardiovascular disease	2(2)	0(0)	0(0)	2(6)		0.334
Other diseases†	0(0)	0(0)	0(0)	0(0)		–
Elicitors						
Antibiotics	48(53)	17(53)	14(58)	17(49)	0.547	0.761
Cephalosporins	31(34)	13(41)	10(42)	8(23)	3.188	0.203
Macrolides	7(8)	1(3)	3(13)	3(9)		0.421
Penicillins	3(3)	0(0)	1(4)	2(6)		0.479
Other antibiotics‡	7(8)	3(9)	0(0)	4(11)		0.282
TCM injection¶	8(9)	2(6)	1(4)	5(14)		0.453
Biologics	7(8)	1(3)	0(0)	6(17)		0.032
Radiocontrast agents	4(4)	0(0)	3(13)	1(3)		0.088
Glucocorticoids	3(3)	0(0)	1(4)	2(6)		0.479
Chemotherapeutic agents	3(3)	1(3)	1(4)	1(3)		1.000
Anesthetics	3(3)	3(9)	0(0)	0(0)		0.057
IV mucosolvan	2(2)	2(7)	0(0)	0(0)		0.189
Antivirus	1(1)	1(3)	0(0)	0(0)		0.615
Vaccine	0(0)	0(0)	0(0)	0(0)	–	–
NSAIDs	0(0)	0(0)	0(0)	0(0)	–	–
Others§	11(12)	4(13)	4(17)	3(9)		0.606

*RTI*, respiratory tract infection; *TCM*, Traditional Chinese medicine; *IV*, intravenous; *NSAIDs*, non-steroid anti-inflammatory drugs

*P values are from Fisher’s exact test or Pearson’s chi-square test of difference across age groups

†Other diseases including 1 patient with epilepsy, 1 patient with cerebral palsy, and 1 patient with short stature

‡Other antibiotics include 3 patients on levofloxacin, 2 patients on vancomycin, 1 patient on lincomycin, and 1 patient on metronidazole

¶The 8 TCM injections are Qingkailing, Shuanghuanglian, Houttuynia, Xiyanping, Xingnaojing, Ligustrazine, and Dehydroandrograpolide (for 2 cases)

§Other medications include calcium dibutyryladenosine cyclophosphate for injection, etamsylate injection, hydroxyethyl starch 200/0.5200/0.5), succinylated gelatin injection, a coenzyme A for injection, levobunolol hydrochloride ophthalmic solution, vitamin K1 injection, colloidal aluminum phosphate gel, fat-soluble vitamin(1) for injection

### Clinical features

The majority of patients with anaphylaxis experienced cardiovascular and respiratory symptoms, and approximately half of the patients developed mucocutaneous manifestations (Table 2). Notably, the 0–5 years group was more likely to develop cyanosis than the 13–17 years group (OR = 5.14, 95%CI [1.74, 15.20], *P* = 0.002), whereas the 13–17 years group were more likely to develop hypotension than the 6–12 years group (OR = 11.79, 95%CI [2.28, 60.87], *P* = 0.002) and neurological symptoms than the 0–5 years group (OR = 3.56, 95%CI [1.26, 10.08], *P* = 0.015). The 13–17 years group was also more likely to develop severe anaphylaxis (OR = 15.46, 95%CI [1.85, 129.33], *P* = 0.002).

**Table 2:** Clinical manifestations during anaphylaxis reactions of children by age group

	All no. (%) (*n* = 91)	0–5 years no. (%) (*n* = 32)	6–12 years no. (%) (*n* = 24)	13–17 years no. (%) (*n* = 35)	*χ* ^2^	*P* *
Mucocutaneous	54(59)	22(69)	14(58)	18(51)	2.092	0.351
Flushing	21(23)	11(34)	4(17)	6(17)	3.551	0.169
Pruritus	16(18)	4(13)	5(21)	7(20)		0.649
Diffuse urticaria	33(36)	13(41)	8(33)	12(34)	0.412	0.814
Angioedema	10(11)	4(13)	3(13)	3(9)		0.837
Conjunctivitis	9(10)	2(6)	3(13)	4(11)		0.742
Respiratory system	66(73)	25(78)	19(79)	22(63)	2.677	0.265
Rhinorrhea	1(1)	0(0)	1(4)	0(0)		0.264
Cough	3(3)	0(0)	0(0)	3(9)		0.111
Voice change	3(3)	0(0)	1(4)	2(6)		0.479
Dyspnea	22(24)	7(22)	7(29)	8(23)	0.517	0.822
Wheezing	21(23)	7(22)	7(29)	7(20)	0.772	0.704
Cyanosis	36(40)	18(56)	11(46)	7(20)	9.724	0.008
Respiratory arrest	2(2)	1(3)	0(0)	1(3)		1.000
Hypoxemia	8(9)	2(6)	4(17)	2(6)		0.368
Cardiovascular system	71(78)	23(72)	15(63)	33(94)	9.476	0.009
Hypotension	69(76)	22(69)	14(58)	33(94)	11.386	0.003
Cardiac arrest	1(1)	0(0)	0(0)	1(3)		1.000
Incontinence	3(3)	0(0)	1(4)	2(6)		0.479
Gastrointestinal system	33(36)	10(31)	10(42)	13(37)	0.663	0.718
Emesis	27(30)	8(25)	9(38)	10(29)	1.060	0.589
Nausea	12(13)	0(0)	5(21)	7(20)		0.010
Abdominal cramping	13(14)	5(16)	5(21)	3(9)		0.382
Diarrhea	0(0)	0(0)	0(0)	0(0)	–	–
Neurological system	35(39)	8(25)	8(33)	19(54)	6.420	0.040
Presyncope	22(24)	6(19)	6(25)	10(29)	0.892	0.640
Syncope	17(19)	6(19)	2(8)	9(26)		0.254
Convulsion	4(4)	1(3)	0(0)	3(9)		0.447
Severity of anaphylaxis						
Mild to moderate	15(17)	10(31)	4(17)	1(3)		0.005
Severe	76(84)	22(69)	20(83)	34(97)		
Outcome						
Death	1(1)	0(0)	0(0)	1(3)		1.000
ICU admission	7(8)	4(13)	2(8)	1(3)		0.325

*ICU*, intensive care unit

*P values are from Fisher’s exact test or Pearson’s chi-square test of difference across age groups

### Analysis of therapies for the treatment of anaphylaxis

A total of 62 patients (68%) received epinephrine for anaphylaxis, but only 49 (54%) received it as an initial treatment. Forty-eight patients had an adequate documentation of administration routes; the number of patients who received IM, SC, IV bolus injection, and IV continuous infusion were 11 (23%), 16 (33%), 15 (31%), and 6 (13%), respectively. Thirty-seven patients had a clear documentation for both the administration route and dosing of epinephrine. A total of 73 (80%) patients received corticosteroids and 68 (75%) received corticosteroid as initial therapy including 34 (37%) on corticosteroid alone and the other 34 (37%) on both corticosteroid and epinephrine. A total 29 (32%) patients received antihistamines, and only 4 (4%) patients received bronchodilators. There was no significant difference among the three different age groups with regards to the route of administration for epinephrine, the route of administration for corticosteroids, or the initial drug therapy of anaphylaxis (Appendix 3).

### Analysis of initial administration routes and initial dosing of epinephrine

A total of 49 patients received epinephrine as initial therapy, including 15 (17%) on epinephrine alone and 34 (37%) on both epinephrine and corticosteroid. Among the 49 patients, data were available from 37 patients with clearly documented initial administration routes of epinephrine and severities of anaphylaxis. The percentage with IM injection, SC injection, IV bolus and IV continuous infusion route was 26, 40, 26, and 9%, respectively. There was no significant difference for number of patients by each administration route (*P* = 0.074). Overall, there was no significant difference in terms of anaphylaxis severity between each administration route (*P* = 0.940). There was a significant difference regarding the frequency of supratherapeutic dosing of epinephrine by each administration route (*χ* ^2^ = 10.407, *P* = 0.005) (Table 3). A supratherapeutic dosing was more likely with the IV bolus route (11/12) compared to IM (5/14, OR = 19.25, 95%CI [1.77, 209.55], *P* = 0.009), and SC (4/11, OR = 19.80, 95%CI [1.94, 201.63], *P* = 0.005). The anaphylaxis severity was not associated with supratherapeutic dosing by an administration route (*χ* ^2^ = 1.481, *P* = 0.512) (Table 3). There was no difference by age in the anaphylaxis treatment and administration for epinephrine.

**Table 3:** Dosing and route of epinephrine administration associated with severity of anaphylaxis

Severity of anaphylaxis	IM injection, no. (%) (*n* = 11)			SC injection, no. (%) (*n* = 14)			IV bolus, no. (%) (*n* = 12)		
	Supratherapeutic dosing	Recommended dosing	*P*	Supratherapeutic dosing	Recommended dosing	*P*	Supratherapeutic dosing	Recommended dosing	*P*
Mild to moderate	1(25)	2(29)	1.000	2(40)	1(11)	0.505	0(0)	1(100)	0.083
Severe	3(75)	5(71)		3(60)	8(89)		11(100)	0(0)	

*P* values are from Fisher’s exact test of difference across age groups. Data were available from 37 patients with clearly documented administration routes (IM injection, SC injection, and IV bolus) and dose of epinephrine. Overall, there is no significant difference regarding the severity of anaphylaxis in each administration route (*P* = 0.512). There was no significant difference between the percentage of severe anaphylaxis between recommended and supratherapeutic dosing groups in each administration route group (*P* value is 1.000, 0.505, and 0.083 for IM, SC, and IV bolus, respectively). Overall, there is a significant difference regarding the overdose rate in each administration route (*P* = 0.005). IV bolus is more likely associated with supratherapeutic dose compared to IM injection (*P* = 0.009) and SC injection (*P* = 0.005). There is no significant difference in overdose rate between IM injection and SC injection (*P* = 1.000)

A total of 20 (54%) patients received supratherapeutic dosing of epinephrine (Table 4). Thirteen patients were overdosed according to the maximum dose of the corresponding administration routes, dosing for 11 patients with IV bolus route exceeded the maximum dose of 0.1 mg, and dosing for 2 patients with IM route exceeded the maximum dose of 0.5 mg. Seven patients under 6 years old were overdosed according to maximum dose of 0.15 mg for this age group (Table 4).

**Table 4:** The information of 20 epinephrine-supratherapeutic dosing pediatric patients

Number	Age (years)	Epinephrine as an initial treatment*	Route of epinephrine	Dose of epinephrine (mg)
1	2.5	Y	IV bolus	0.50
2	4.0	Y	IV bolus	0.50
3	4.2	Y	IV bolus	3.00
4	9.0	N	IV bolus	0.50
5	10.0	N	IV bolus	0.30
6	10.2	Y	IV bolus	1.00
7	13.0	Y	IV bolus	0.50
8	14.2	N	IV bolus	0.50
9	14.9	N	IV bolus	0.50
10	15.1	N	IV bolus	1.00
11	17.9	Y	IV bolus	0.20
12	4.0	Y	IM	0.20
13	4.7	Y	IM	0.33
14	8.5	N	IM	1.00
15	17.2	Y	IM	1.00
16	2.0	Y	SC	0.30
17	3.7	Y	SC	0.50
18	3.8	Y	SC	0.30
19	4.0	Y	SC	0.30
20	5.8	Y	SC	0.40

*Y, epinephrine as an initial treatment; N, epinephrine not as an initial treatment

Among 91 cases, only 1 patient died and this patient was treated initially with epinephrine; 7 patients were admitted to ICU, of those 4 patients received epinephrine (2 were initially treated) and 2 were treated with supratherapeutic doses. There was no significant difference regarding outcome between patients who received epinephrine as an initial treatment and those who did not (*χ* ^2^ = 4.437, *P* = 0.229, Appendix 4). Overall, our analysis showed there were no significant association between symptoms and use of epinephrine, whether use epinephrine as an initial treatment, or administration route of epinephrine (Appendix 5–7).

## Discussion

Using the BPD data, our study is the first to analyze the clinical features and treatment of pediatric DIA patients in clinical settings over a decade in Beijing, China. A total of 91 pediatric cases were identified and assessed, accounting for 7.7% (91/1189) of both adult and pediatric DIA patients. Antibiotics, TCM injections and biologics were the top three drug triggers. The most common anaphylactic clinical features were manifestations of the cardiovascular and respiratory systems, and children may present signs and symptoms differently based on age groups. Epinephrine is underutilized, and only 54% receive it as the initial treatment. Only 23% of patients received epinephrine by the IM route, suggesting the administration route is not appropriate. The percentage of supratherapeutic dose of epinephrine is high, 92% (11/12) for the IV bolus, and 36% (5/14) for IM, and 36% (4/11) for SC. Furthermore, the overdose frequency of IV bolus was significantly higher than both IM and SC combined.

### Demographics and elicitors of pediatric patients with DIA

The higher frequency for males in the 0–5 year and the 13–17 year age groups is consistent with the data from the European anaphylaxis registry [10] reporting that boys are more likely to experience anaphylaxis. Infectious diseases, stress/operation/trauma and tumor were the principal admission diagnoses; disease distribution was similar among the three age groups. Although asthma is considered to be one of the important risk factors for anaphylaxis [9, 15], there was only one case with a current asthma diagnosis in our study, possibly related to our small sample size or the relatively low prevalence of asthma in China [22].

Antibiotics were the most common trigger, which is in line with a few previous studies indicating antibiotics are the most common cause of DIA [9, 19, 23]. Our study suggests that clinical signs and symptoms related to anaphylaxis should be closely monitored when children are administered antibiotics in the hospital setting, particularly β-lactams. Unlike the US or the European reports, TCM injections were the next most common cause of DIA, including *Qingkailing*, *Shuanghuanglian*, *Houttuynia*, etc., which is consistent with a recent study from China [16]. TCM injections have relatively complex formulations and may cause anaphylaxis [5, 16], thus they should be avoided if possible. Although non-steroidal anti-inflammatory drugs (NSAIDs) are important triggers for DIA in adults [1, 6], our analysis did not observe any NSAID-induced anaphylaxis, which is consistent with a previous study reporting that NSAIDs were infrequent causes of pediatric anaphylaxis [19].

### Target organ involvement and clinical manifestations during anaphylaxis reactions of DIA children

The two most frequent clinical features of DIA in our study were overall cardiovascular and respiratory manifestations, followed by mucocutaneous symptoms. Although mucocutaneous symptoms are generally considered as a typical sign of allergy, our study implies that absence of such symptoms might not be sufficient to exclude the diagnosis of anaphylaxis. Among three age groups, we observed significant differences for some symptoms (neurological systems, cyanosis, hypotension, and nausea) and anaphylaxis severity, which is in line with previous studies that observed age-related patterns in the clinical presentation of pediatric anaphylaxis [10, 24]. Considering age differences regarding DIA, symptoms could facilitate early recognition and prompt treatment of this potentially life-threatening condition.

The present study found that 36% of DIA children had gastrointestinal manifestations, with emesis and abdominal cramping as the most common symptoms. This is consistent with a previous study [29], suggesting that gastrointestinal symptoms should be assessed when considering DIA, and abdominal cramping should especially arouse concern in 0–5-year-old children because they may not accurately express this discomfort.

### Epinephrine administration and pediatric DIA

Similar to our study, the underuse of epinephrine has also been described by previous studies [3, 30]. As the first-line treatment in anaphylaxis, epinephrine is recommended by primary guidelines [21, 26, 28]. The low frequency of epinephrine utilization and not using it as the initial therapy in our study could be related to the failure to recognize anaphylaxis initially or the perceived severity of adverse reactions associated with the use of epinephrine. Clinicians should use epinephrine as a first-line therapy as soon as they make a diagnosis of anaphylaxis since delayed epinephrine administration is associated with a risk of hospitalization and poor outcomes [2, 8].

Our study showed a higher percentage of IV injection (bolus and continuous infusion) but lower intramuscular use. More than two thirds of the administration route was by IV bolus. Studies have found that epinephrine administered by the IM route achieves peak concentrations faster than that given by subcutaneous injection, and IM is also safer than the IV bolus injection [4, 27]. IV continuous infusion of epinephrine should be preferred when anaphylaxis patients do not respond to repeated IM injections [21]. The underuse of the IM administration may be because that physicians do not recognize that IM is the optimal route in anaphylaxis treatment, thus further education is needed.

This study showed that the overdosing frequency of epinephrine is quite high, especially with the IV bolus route, which was consistent with a recent study [4]. Among the 20 supratherapeutic dosing patients, 10 were 0–5 years old, and 13 patients were administered epinephrine exceeding the recommended maximum dose, especially with the IV bolus route. This high frequency of supratherapeutic dosing may be due to (1) the overuse of IV bolus injection, (2) the perceived severity of the anaphylaxis signs and symptoms, (3) physicians not knowing the recommended maximum dose of epinephrine in treating anaphylaxis, especially for the 0–5 year age group, and (4) confusion about the recommended dose treating anaphylaxis with the dose treating cardiac arrests. The fact that only one epinephrine formulation (1:1000, 1 mg/ml) is available in China may also contribute to the high frequency of overdosing.

IV bolus administration of epinephrine should be avoided whenever possible due to the risk of cardiac arrhythmias and potential for inappropriate dosing [4]. The epinephrine dosing by IV bolus injection in anaphylaxis is also significantly lower than the dose recommended for cardiac arrest (1 mg of 1:10,000) IV bolus [7]. Caution should be exercised when treating the 0–5-year-old age group especially with an IV bolus as overdosing tends to occur more frequently based on our analysis.

### Other common treatments in pediatric patients with DIA

Our study observed a higher frequency of corticosteroids use compared to epinephrine use despite corticosteroids is recommended only as second-line adjunctive therapy [21, 26, 28]. Additionally, when used as the only initial monotherapy, corticosteroid use was approximately two times higher than epinephrine. This discrepancy between practice and guidelines [21, 26, 28] may be because physicians fail to recognize that epinephrine should be the cornerstone and first-line therapy in anaphylaxis.

Although wheezing and dyspnea symptoms each accounted for about 20% of anaphylaxis in children, only 4% of them were administered inhaled short-acting beta-2 agonists. This suggests that β2-agonist inhalation treatment should be enhanced in anaphylaxis management in order to quickly alleviate lower respiratory tract symptoms. Furthermore, although a previous study showed that the combination of systemic H1- and H2-antihistamines are more beneficial than H1-antihistamine monotherapy in relieving some cutaneous symptoms in those experiencing acute allergic reactions [18], only 1 of our patients received H2-antihistamines.

### Limitations

The study has several limitations: (1) the analysis was based on self-reported cases by health care professionals from the BPD, which may include incomplete data; (2) we may not include all DIA pediatric patients: cases missed if clinicians did not report using the terms related to allergy or anaphylaxis or hypersensitivity; and (3) the sample size was small. The method we have taken should be robust against a range of potential biases: rigorous inclusion/exclusion criteria were utilized and all potential anaphylaxis cases were adjudicated by trained physician/allergists; and only patients with complete data record were included in the analysis.

The present study showed there were differences in clinical symptoms and severities of DIA among 0–5, 6–12, and 13–17-year-old patients. For DIA in pediatric patients in the Beijing area, we found that the three common triggers were antibiotics, TCM injections, and biologics. Epinephrine was underused compared to corticosteroids in the treatment of anaphylaxis. IV bolus of epinephrine was overused and was associated with supratherapeutic dosing.

## Electronic supplementary material

ESM 1 (DOCX 902 kb).

## Abbreviations

DIA: Drug-induced anaphylaxis
ED: Emergency department
IM: Intramuscular
SC: Subcutaneous
IV: Intravenous
BPD: Beijing Pharmacovigilance Database
OR: Odds ratio
CI: Confidence intervals
TCM: Traditional Chinese medicine
