# Supplementary material for: Clinical features and treatment of pediatric patients with drug-induced anaphylaxis: a study based on pharmacovigilance data
Source: Eur J Pediatr. 2017 Nov 22;177(1):145–54. doi: 10.1007/s00431-017-3048-z (PMC5748398; doi:10.1007/s00431-017-3048-z)
Supplement: Supplementary file 1 — (DOCX 902 kb). [file 431_2017_3048_MOESM1_ESM.docx]

**Supplementary Appendix**

**Supplement to:**

**Clinical features and treatment of pediatric patients with drug-induced anaphylaxis: a study based on pharmacovigilance data**

**Supplementary Materials**

Contents

**Appendix 1 – Definition and severity of anaphylaxis**

**Appendix 2 – Flowchart of patient selection**

**Appendix 3 – Table s1 Treatment of anaphylaxis by age group**

**Appendix 4 – Table s2 The outcome of patients based on whether epinephrine was administered as an initial treatment**

**Appendix 5 – Table s3 The percentage of epinephrine treatment with different symptoms**

**Appendix 6 – Table s4 The percentage of epinephrine as an initial treatment with different symptoms**

**Appendix 7 – Table s5 The route of administration of epinephrine based on different symptoms**

**Appendix 1** – **Definition and severity of anaphylaxis**

**Clinical criteria for diagnosing anaphylaxis**

Anaphylaxis is highly likely when any one of the following three criteria is fulfilled:

**1. Acute onset of an illness (minutes to several hours) with involvement of the skin, mucosal tissue, or both (e.g., generalized hives, pruritus or flushing, swollen lips–tongue–uvula AND AT LEAST ONE OF THE FOLLOWING**

a. Respiratory compromise (e.g., dyspnea, wheeze–bronchospasm, stridor, reduced PEF, hypoxemia)

b. Reduced BP or associated symptoms of end-organ dysfunction (e.g., hypotonia [collapse], syncope, incontinence)

**2. Two or more of the following that occur rapidly after exposure to a likely allergen for that patient (minutes to several hours):**

a. Involvement of the skin–mucosal tissue (e.g., generalized hives, itch-flush, swollen lips–tongue–uvula)

b. Respiratory compromise (e.g., dyspnea, wheeze–bronchospasm, stridor, reduced PEF, hypoxemia)

c. Reduced BP or associated symptoms (e.g., hypotonia [collapse], syncope, incontinence)

d. Persistent gastrointestinal symptoms (e.g., crampy abdominal pain, vomiting)

**3. Reduced BP after exposure to known allergen for that patient (minutes to several hours):**

a. Infants and children: low systolic BP (age specific) or >30% decrease in systolic BP*

b. Adults: systolic BP of <90 mmHg or >30% decrease from that person’s baseline

**Notes**

PEF, peak expiratory flow; BP, blood pressure.

Reproduced from Sampson et al. (17) with permission (C).

*Low systolic blood pressure for children is defined as <70 mmHg from 1 month to 1 year, less than (70 mmHg + [2×age]) from 1 to

10 years and <90 mmHg from 11 to 17 years.

Reference: Sampson HA, Muñoz-Furlong A, Campbell RL, et al. Second symposium on the definition and management of anaphylaxis: summary report — Second National Institute of Allergy and Infectious Disease/Food Allergy and Anaphylaxis Network symposium. J Allergy Clin Immunol 2006; 117:391-7.

**Definition for Severity of Anaphylaxis**

***Mild to moderate anaphylaxis.*** Patients with any of the findings listed for acute allergic reaction with additional respiratory, cardiovascular, gastrointestinal, or neurologic features or presenting with any of these additional features alone in the setting of an allergic reaction. The additional features included a history of shortness of breath or dyspnea, wheeze, hoarseness, and nausea or vomiting. Physical findings recorded on arrival in the ED or within 30 minutes of arrival included the presence of bronchospasm, systolic blood pressure >90 mmHg, respiratory rate <25/min, and a normal Glasgow Coma Scale score.

***Severe anaphylaxis.*** Patients with any of the findings listed for mild to moderate anaphylaxis but with potentially life-threatening symptoms or signs. These included any one or more of the following: history of loss of consciousness, syncope, or dizziness or light-headedness at any time; systolic blood pressure on arrival in the ED or within 30 minutes of arrival of <90 mmHg; a Glasgow Coma Scale score on arrival or within 30 minutes of arrival of <15, related to cardiovascular system collapse and/or neurologic dysfunction from hypoperfusion, hypoxia, and the direct effect of mediators. Also included were patients with any 1 or more of the following: history of shortness of breath, wheeze, hoarseness or bronchospasm plus any 1 or more of stridor, cyanosis, laryngeal edema or a respiratory rate ≥25/min on ED arrival or within 30 minutes from respiratory system dysfunction.

Note: Low systolic blood pressure for children is defined as <70 mmHg from 1 month to 1 year, less than (70 mmHg + [2×age]) from 1 to 10 years and <90 mmHg from 11 to 17 years.

Reference: Brown AF, McKinnon D, Chu K. Emergency department anaphylaxis: a review of 142 patients in a single year. J Allergy Clin Immunol 2001; 108(5):861–6.

**Appendix 2 – Flowchart of patient selection**


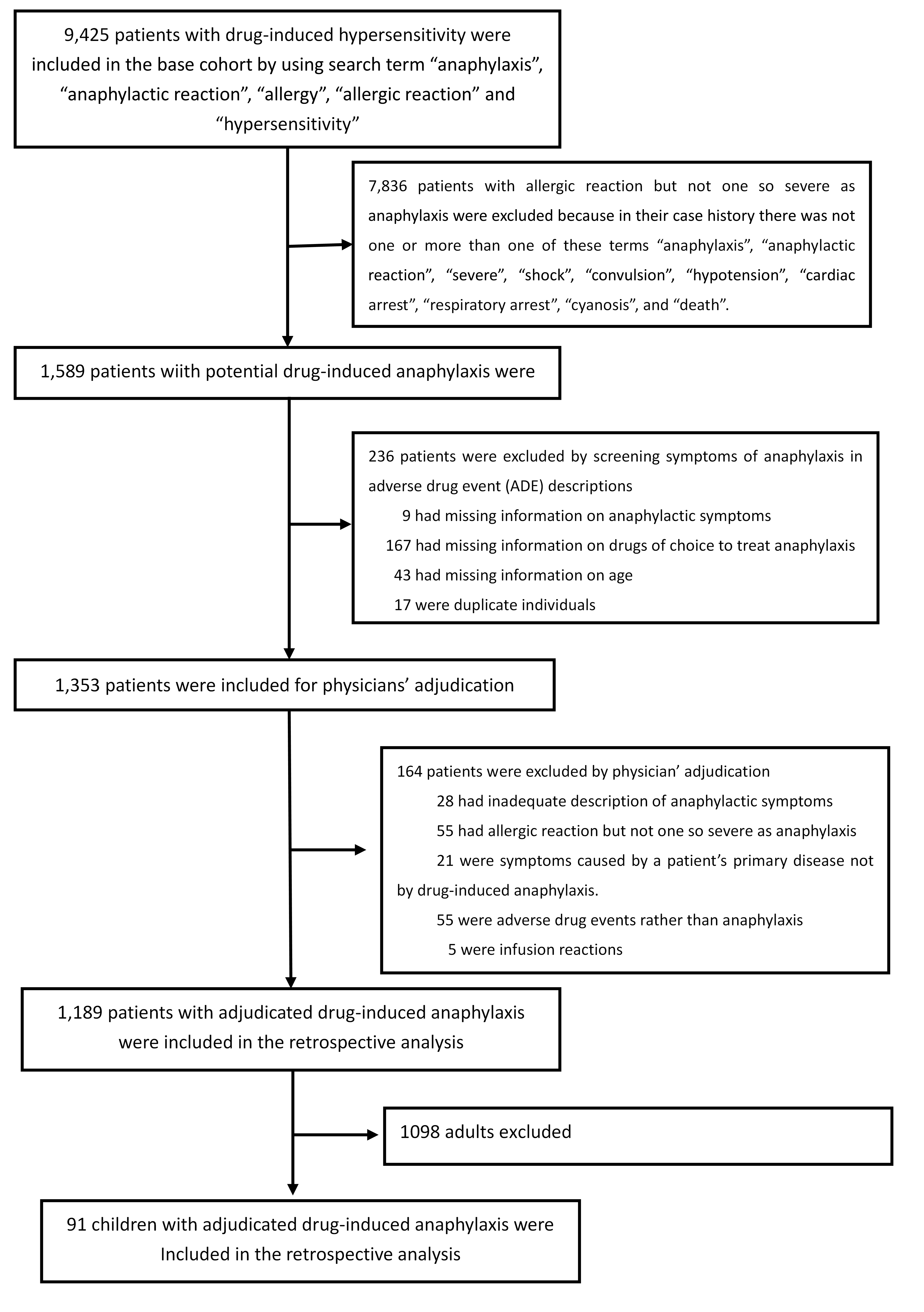


**Appendix 3 -** Table s1 Treatment of anaphylaxis by age group

|  | ALL  No. (%)  (n=91) | 0-5 years  No. (%)  (n=32) | 6-12 years  No. (%)  (n=24) | 13-17 years  No. (%)  (n=35) | χ^2^ | P * |
| --- | --- | --- | --- | --- | --- | --- |
| **Epinephrine** | 62(68) | 22(69) | 17(71) | 23(66) | 0.181 | 0.914 |
| **Epinephrine as initial treatment** | 49(54) | 17(53) | 13(54) | 19(54) | 0.010 | 0.995 |
| **Administration route** ** | 48 | 19 | 13 | 16 |  |  |
| Intramuscular | 11(23) | 3(16) | 4(31) | 4(25) |  | 0.628 |
| Intravenous | 21(44) | 7(37) | 7(54) | 7(44) | 0.907 | 0.635 |
| Bolus | 15(31) | 5(26) | 4(31) | 6(38) |  | 0.922 |
| Continuous infusion | 6(13) | 2(11) | 3(23) | 1(6) |  | 0.413 |
| Subcutaneous | 16(33) | 9(47) | 2(15) | 5(31) |  | 0.170 |
| **Epinephrine Administration dosage (mg) †** | 37 | 16 | 8 | 13 |  |  |
| Intramuscular | 0.33(0.10, 1.00) | 0.20(0.10, 0.33) | 0.24(0.10,1.00) | 0.50(0.50,1.00) | 4.409 | 0.110 |
| IV bolus | 0.50(0.20, 3.00) | 0.50(0.50, 3.00) | 0.50(0.30,1.00) | 0.50(0.20,1.00) | 0.589 | 0.745 |
| Subcutaneous | 0.30(0.10, 0.50) | 0.30 (0.10, 0.50) | 0.30(0.30,0.30) | 0.50(0.20, 0.50) | 3.122 | 0.210 |
| **Dosing of epinephrine ‡** |  |  |  |  |  |  |
| Recommended dose | 17(46) | 6(38) | 4(50) | 7(54) |  | 0.694 |
| Supratherapeutic dose | 20(54) | 10(63) | 4(50) | 6(46) |  |  |
| **Corticosteroid** | 73(80) | 24(75) | 20(83) | 29(83) | 0.837 | 0.674 |
| **Administration route§** |  |  |  |  |  |  |
| Intravenous | 51(56) | 19(59) | 10(42) | 22(63) | 2.817 | 0.244 |
| Intramuscular | 9(10) | 1(3) | 4(17) | 4(11) |  | 0.238 |
| Oral | 3(3) | 2(6) | 0(0.0) | 1(3) |  | 0.622 |
| **Epinephrine + Corticosteroid** | 48(53) | 17(53) | 13(54) | 18(51) | 0.046 | 1.000 |
| **Bronchodilator** | 4(4) | 2(6) | 2(8) | 0(0) |  | 0.184 |
| β2-agonist (inhalation) | 4(4) | 2(6) | 2(8) | 0(0) |  | 0.184 |
| **Antihistamine** | 29(32) | 9(28) | 10(42) | 10(29) | 1.443 | 0.511 |
| **Administration route¶** |  |  |  |  |  |  |
| Intravenous | 5(6) | 2(6) | 2(8) | 1(3) |  | 0.627 |
| Intramuscular | 14(15) | 5(16) | 3 (13) | 6(17) |  | 0.935 |
| Oral | 8(9) | 1(3) | 4(17) | 3(9) |  | 0.234 |
| **Categories** |  |  |  |  |  |  |
| H1 blocker | 29(32) | 9(28) | 10(42) | 10(29) | 1.443 | 0.486 |
| H2 blocker | 1(1) | 0(0) | 0(0) | 1(3) |  | 1.000 |

*Note:*

*P values are from Fisher’s exact test or Pearson’s chi-square test of difference across age groups.

** 48 patients had a clear record of epinephrine administration route.

† 37 patients had a clear record for both administration route and dosing of epinephrine.

‡ 37 patients had a clear record of epinephrine dosage and sufficient data to judge whether overdose occurred.

§ 63 patients had a clear record of administration route for corticosteroids.

¶ 29 patients had a clear record of administration route of antihistamines.

**Appendix 4 -**Table s2 The outcome of patients based on whether epinephrine was administered as an initial treatment

| Epinephrine as an initial treatment | | Outcome | | | |
| --- | --- | --- | --- | --- | --- |
|  |  | Recovery | Improvement | No improvement | Death |
|  | Yes | 29 | 20 | 0 | 1 |
|  | No | 5 | 6 | 1 | 0 |
| Total | | 34 | 26 | 1 | 1 |

Notes:

There was no significant difference regarding outcome between patients who received epinephrine as an initial treatment and those who did not receive epinephrine as an initial treatment (χ2=4.437, P=0.229). The hypothesis testing is done by Fisher’s exact test.

**Appendix 5 -** Table s3 The percentage of epinephrine treatment with different symptoms

| Symptom | Epinephrine No. (%) | | χ2 | p |
| --- | --- | --- | --- | --- |
|  | Yes(n=62) | No(n=29) |  |  |
| Cardiovascular | 46(74) | 25(86) | 1.663 | 0.197 |
| Respiratory | 45(73) | 21(72) | 0.000 | 0.987 |
| Mucocutaneous | 37(60) | 17(59) | 0.009 | 0.924 |
| Gastrointestinal | 25(40) | 8(28) | 1.387 | 0.239 |
| Central nervous system | 23(37) | 12(41) | 0.153 | 0.696 |

*Note:*

*P values are from Pearson’s chi-square test of difference between patients who received epinephrine as a treatment and those who did not receive epinephrine as a treatment

**Appendix 6 -** Table s4 The percentage of epinephrine as an initial treatment with different symptoms

| Symptom | Epinephrine as first line No. (%) | | χ2 | p |
| --- | --- | --- | --- | --- |
|  | Yes(n=15) | No(n=76) |  |  |
| Cardiovascular | 9(60) | 62(82) | 2.260 | 0.133 |
| Respiratory | 8(53) | 58(76) | 2.268 | 0.132 |
| Gastrointestinal | 8(53) | 25(33) | 2.264 | 0.132 |
| Mucocutaneous | 7(47) | 47(62) | 1.196 | 0.274 |
| Central nervous system | 5(33) | 30(40) | 0.200 | 0.655 |

*Note:*

*P values are from Pearson’s chi-square test of difference between patients who received epinephrine as an initial treatment and those who did not receive epinephrine as an initial treatment.

**Appendix 7 -** Table s5 The route of administration of epinephrine based on different symptoms

| Symptom | IM injection,  No. (%) (n=11) | SC injection,  No. (%) (n=16) | IV bolus,  No. (%) (n=15) | Continuous infusion  No. (%) (n=6) | χ2 | p* |
| --- | --- | --- | --- | --- | --- | --- |
| Respiratory | 8(73) | 13(81) | 10(67) | 3(50) | - | 0.544 |
| Mucocutaneous | 7(64) | 13(81) | 9(60) | 2(33) | - | 0.221 |
| Cardiovascular | 6(55) | 10(63) | 12(80) | 5(83) | - | 0.453 |
| Central nervous system | 5(46) | 7(44) | 4(27) | 2(33) | - | 0.728 |
| Gastrointestinal | 4(36) | 5(31) | 8(53) | 3(50) | - | 0.625 |

*Notes:*

*P values are from Fisher’s exact test of difference across route groups.
